# Synthesis of a New Series of *N,N’-*Dimethyltetrahydrosalen(H_2_ [H_2_Me]salen) Ligands by the Reductive Ring-Opening of 3,3'-Ethylene-bis(3,4-dihydro-6-substituted-2*H*-1,3-benzoxazines) 

**DOI:** 10.3390/molecules15064102

**Published:** 2010-06-07

**Authors:** Augusto Rivera, Jicli José Rojas, Jairo Salazar-Barrios, Mauricio Maldonado, Jaime Ríos-Motta

**Affiliations:** Departamento de Química, Universidad Nacional de Colombia, Bogotá, Colombia, A.A. 14490, Colombia

**Keywords:** tetrahydrosalen, salan, bis-benzoxazines, tetradentate ligand

## Abstract

A new series of *N*,*N*'-bis(2'-hydroxy-5'-substituted-benzyl)-*N*,*N*´-dimethylethane-1,2-diamines (*N,N'*-dimethyltetrahydrosalen) ligands were prepared in good yield by reduction of the respective 3,3'-ethylene-bis(3,4-dihydro-6-substituted-2*H*-1,3-benzoxazine) precursors with sodium borohydride . The ligands were characterized by IR, NMR, and elemental analysis, which showed the compounds to be consistent with the proposed structures. Ring-opening reactions of bis-1,3-benzoxazines in the presence of sodium borohydride to produce *N,N’-*dimethylated tetrahydrosalens (H_2_ [H_2_Me]salen) have not been reported in the literature.

## 1. Introduction

**Figure 1:**
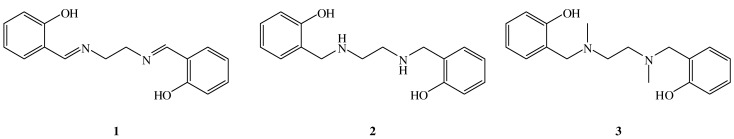
Structures of compounds **1-3**.

The salen-type class of ligands (H_2_ salen; *N*,*N’*-disalicylidene-1,2-diaminoethane; **1**, Figure 1) has had an extensive and continuing history in transition metal chemistry. Hydrogenation of the imine bond of salen compounds produces a new tetradentate ligand, which is known generally as salan (H2[H4]salen; tetrahydrosalen; *N*,*N’*-bis(2-hydroxybenzyl)-1,2-diaminoethane; **2**, Figure 1) [1]. While the salen ligands feature two sites capable of covalent bonding with an electropositive element, the H_4_ salan ligands contain four such sites, and are therefore ideally suited to bind multiple metals [2].

Tetrahydrosalen-type ligands are intimately involved with a number of metal coordination complexes, which include those elements located in groups 12, 13 and 14 [3]. Some of them have been mostly studied in polymerization catalysis in the past ten years [4,5,6]. Interest in these tetradentate ligands, whose properties may be manipulated by changing the bridging unit between the two nitrogen atoms, the substituents on the amine group, or the substitution patterns on the phenols, has stimulated research efforts in developing synthetic procedures to obtain a variety of these compounds [7,8,9,10,11,12,13,14,15]. Tetrahydrosalen, *N*,*N’*-dimethylated tetrahydrosalen **3** and its derivatives have rarely been studied, and the most common approach for the preparation of this class of compounds has involved the isolation of the salan intermediate followed by additional substitution steps on the salan products [16,17], or condensation of salans with formaldehyde/acetic acid followed by *in situ* sodium borohydride reduction to give the *N*-methylated salans [18]. Other procedures employ the reductive amination of *N*,*N*'-dimethylethylene diamine with NaBH_3_(CN) [19,20]. Recently, Tshuva *et al.* [14] reported a single-step synthetic procedure enabling the preparation in high yield of a variety of salan compounds, including *N*,*N*-disubstituted salans, by a Mannich condensation of substituted phenols, formaldehyde and *N,N’*-substituted-diamines. In a series of earlier works, we reported on the successful synthesis of 3,3'-ethylene-bis(3,4-dihydro-6-substituted-2*H*-1,3-benzoxazines) (BISBOAs) thru the condensation of *p*-substituted phenols, formaldehyde and ethylenediamine [21,22,23]. Herein, we report on the usefulness of these compounds for the expedient synthesis of a new series of *N*,*N’*-dimethylated tetrahydrosalens.

Based on a comparison of the basicity of tetrahydrosalen and salen, where the basicity decreases, we expected that the methyl functionality in tetrahydrosalens would provide the best template for metal binding. On the other hand, it is well known that tetrahydrosalen associated with metal centers displays *cis*-octahedral coordination geometry, which can form two possible diastereomers (cis *fac-mer* and cis *fac-fac*) [24]. Each of these can exist as a pair of chiral-at-metal enantiomers [8].

## 2. Results and Discussion

**Scheme 1:**
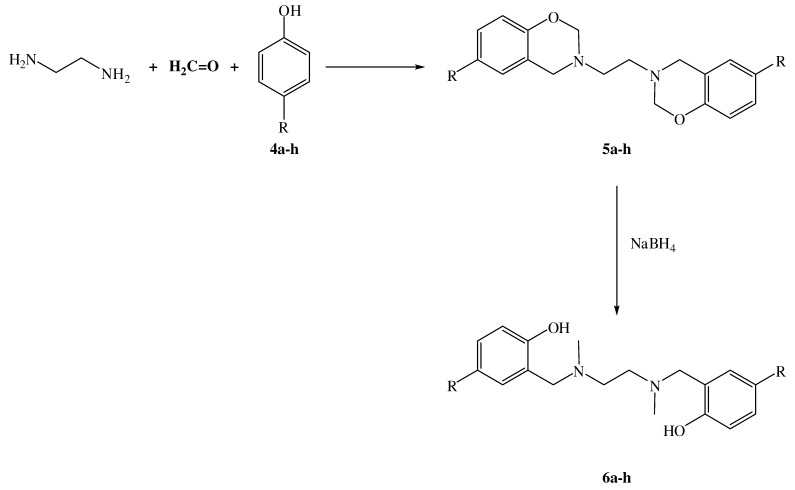
Synthesis of the *N,N*´-dimethylsalan from the BISBOAs.

The overall procedure for the preparation of *N*,*N*'-bis(2'-hydroxy-5'-substituted-benzyl)-*N*,*N*´-dimethylethane-1,2-diamines **6a-h** is depicted in Scheme 1. The 3,3'-ethylene-bis(3,4-dihydro-6-substituted-2*H*-1,3-benzoxazines) **5a-h** used were prepared according to a previously reported procedure [21,22,23] that involves a one-pot condensation–cyclization reaction of the appropriate phenol **4a-h** with an excess of 37% aqueous formaldehyde and ethylenediamine in a mixture of dioxane and water. 

**Scheme 2:**
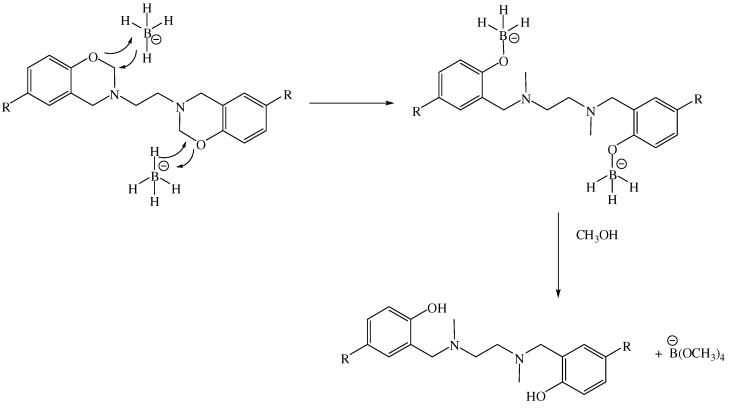
Mechanism of reduction of **5a-h** with NaBH_4_.

Based on previous results reported for the reduction of naphtho-1,3-oxazines [25] and benzo-1,3-oxazines we anticipated that the reaction between compounds **5a-h** and sodium borohydride would yield tetrahydrosalens **6a-h** [26]. Additionally, the efficacy of sodium borohydride as a reducing agent should give the expected tetrahydrosalen products. In fact, the reduction with sodium borohydride of the appropriate BISBOAs (**5a-h**) to the respective *N,N'*-dimethylated tetrahydrosalens occurs readily and with good yields, ranging from 36% to 70% (Table 1). The structures of all the synthesized molecules were confirmed by elemental analysis and spectral (FT-IR, ^1^H-NMR, ^13^C-NMR) data. The FT-IR spectra of compounds **6a-h** lack the characteristic absorption peaks of the O-CH_2_-N methylene group of the benzoxazine ring structure at 1,226 cm^-1^ (asymmetric stretching of C–O–C) and 1,035 cm^-1^ (symmetric stretching of C–O–C). The spectra did show, however, the presence of a OH group with absorptions near 3,400 cm^-1^. In the ^1^H-NMR, characteristic peaks of the 1,3-oxazine ring were not observed at *ca.* 5.0 ppm, but a new two-methyl singlet (6H) appeared with a chemical shift range of 2.21–2.30 ppm. This indicates that the double reduction of **5a-h** with NaBH_4_ proceeds by the chemoselective cleavage of the O-CH_2_ bond of the *N,O*-acetal moiety of BISBOAs. This chemoselectivity may be related to the preference of the boron atom toward alkoxy complex formation, which is more favorable to a subsequent hydrolysis reaction than the aminoborane obtained by reductive cleavage of the CH_2_-N bond. A reduction mechanism in two steps is proposed in Scheme 2.

**Table 1:** Substrate scope of reduction of BISBOAs.

Entry	Compound	R	Product	Yield (%)
1	**5a**	F	**6a**	38
2	**5b**	Cl	**6b**	61
3	**5c**	Br	**6c**	40
4	**5d**	I	**6d**	59
5	**5e**	COOMe	**6e**	36
6	**5f**	COOEt	**6f**	66
7	**5g**	COOPr	**6g**	70
8	**5h**	COOBu	**6h**	68

## 3. Experimental

### 3.1. General

The 3,3'-ethylene-bis(3,4-dihydro-6-substituted-2*H*-1,3-benzoxazines) **5a-h** used were prepared according to the literature procedure [21,22,23]. Chemicals were used without further purification, and infrared spectra were recorded on a Perkin–Elmer FT-IR Paragon spectrometer with a KBr disk. ^1^H- and ^13^C-NMR spectra were measured on a Bruker Advance 400 MHz spectrometer in CDCl_3_ operating at 400 MHz and 100 MHz, respectively. Elemental analyses (C, H, N) were determined in a Carlo-Erba model 1106 analyzer. Melting points (uncorrected) were determined on an Electrothermal 9100 melting point apparatus.

### 3.2. General procedure for synthesis of BISBOAs

To a stirred and cooled solution of formaldehyde 37% (2.8 mL, 37.4 mmol) in dioxane (40 mL) is added slowly dropwise ethylenediamine (0.65 mL, 9.36 mmol). After stirring for 15 min at 5 ºC a solution of respectively 4-substituted-phenol (18.7 mmol) in dioxane (17 mL) is added dropwise with stirring. The mixture is gently refluxed for 4–24 h. After cooling to room temperature, the solvent is removed *in vacuo* and the crude product is recrystallized from methanol.

*3,3'-ethylene-bis(3,4-dihydro-6-chloro-2H-1,3-benzoxazine)* (**5b**). m.p. 170.2 ºC (literature [22]: 170–173 ºC). ^1^H-NMR δ (ppm): 3.02 (s, 4H, NCH_2_CH_2_N), 3.99 (s, 4H, Ar-CH_2_-N), 4.87 (s, 4H, O-CH_2_-N), 6.75 (d, 2H, *J* = 8.66 Hz, Ar-H), 6.94 (d, 2H, *J* = 2.4 Hz, Ar-H), 7.10 (dd, 2H, *J* = 8.66 Hz, *J* = 2.4 Hz, Ar-H). ^13^C-NMR δ (ppm): 49.6, 50.3, 82.8, 117.9, 121.4, 125.3, 127.2, 127.8, 152.7. The spectral data were consistent with literature values [22].

*3,3'-ethylene-bis(3,4-dihydro-6-bromo-2H-1,3-benzoxazine)* (**5c**). m.p. 177.2 ºC (literature [23]: 178–179 ºC). ^1^H-NMR δ (ppm): 2.91 (s, 4H, NCH_2_CH_2_N), 3.98 (s, 4H, Ar-CH_2_-N), 4.85 (s, 4H, O-CH_2_-N), 6.65 (d, 2H, *J* = 8.66 Hz, Ar-H), 7.06 (d, 2H, *J* = 2.4 Hz, Ar-H), 7.19 (dd, 2H, *J* = 8.66 Hz, *J* = 2,4 Hz, Ar-H). ^13^C-NMR δ (ppm): 49.4, 51.2, 82.1, 110.6, 117.7, 121.0, 129.9, 131.4, 153.6. The spectral data were consistent with literature values [23].

*Dimethyl 3,3'-(ethane-1,2-diyl)bis(3,4-dihydro-2H-benzo[e][1,3]oxazine-6-carboxylate)* (**5e**). m.p. 152.2 ºC (literature [22]: 151–154 ºC). ^1^H-NMR δ (ppm): 2.96 (s, 4H, NCH_2_CH_2_N), 3.89 (s, 6H, CH_3_-O), 4.06 (s, 4H, Ar-CH_2_-N), 4.97 (s, 4H, O-CH_2_-N), 6.77 (d, 2H, *J* = 8.4 Hz, Ar-H), 7.69 (d, 2H, *J* = 2.0 Hz, Ar-H), 7.83 (dd, 2H, *J* = 8.4 Hz, *J* = 2.0 Hz, Ar-H). ^13^C-NMR δ (ppm): 49.6, 50.2, 51.8, 83.3, 116.4, 119.4, 122.8, 129.6, 129.7, 158.2, 162.2. The spectral data were consistent with literature values [22].

*Diethyl 3,3'-(ethane-1,2-diyl)bis(3,4-dihydro-2H-benzo[e][1,3]oxazine-6-carboxylate)* (**5f**). m.p. 142.5 ºC. ^1^H-NMR δ (ppm): 1.38 (t, *J =* 8.00 Hz, 6H, **CH_3_**-CH_2_-O), 2.95 (s, 4H, NCH_2_CH_2_N), 4.07 (s, 4H, Ar-CH_2_-N), 4.24 (q, *J =* 8.00 Hz, 4H, CH_3_-**CH_2_**-O), 4.97 (s, 4H, O-CH_2_-N), 6.78 (d, 2H, *J* = 8.4 Hz, Ar-H), 7.69 (d, 2H, *J* = 2.0 Hz, Ar-H), 7.81 (dd, 2H, *J* = 8.4 Hz, *J* = 2.0 Hz, Ar-H). ^13^C-NMR δ (ppm): 13.6, 49.6, 50.3, 51.8, 83.3, 116.4, 119.4, 122.8, 129.6, 129.7, 158.2, 162.2.

*Dipropyl 3,3'-(ethane-1,2-diyl)bis(3,4-dihydro-2H-benzo[e][1,3]oxazine-6-carboxylate)* (**5g**). m.p. 127.2–127.9 ºC. ^1^H-NMR δ (ppm): 1.02 (t, *J =* 7.5 Hz, 6H, **CH_3_**-CH_2_-O), 1.79 (m, *J =* 7.5 Hz, 4H, CH_3_-**CH_2_**-CH_2_-O), 2.95 (s, 4H, NCH_2_CH_2_N), 4.07 (s, 4H, Ar-CH_2_-N), 4.22 (t, *J =* 7.6 Hz, 4H, CH_3_-CH_2_-**CH_2_**-O), 4.95 (s, 4H, O-CH_2_-N), 6.78 (d, 2H, *J* = 8.4 Hz, Ar-H), 7.69 (d, 2H, *J* = 2.0 Hz, Ar-H), 7.81 (dd, 2H, *J* = 8.4 Hz, *J* = 2.0 Hz, Ar-H). ^13^C-NMR δ (ppm): 10.5, 22.2, 49.6, 50.3, 53.8, 83.3, 116.4, 119.4, 122.8, 129.6, 129.7, 158.2, 162.2.

*Dibutyl 3,3'-(ethane-1,2-diyl)bis(3,4-dihydro-2H-benzo[e][1,3]oxazine-6-carboxylate)* (**5h**). m.p. 89.2 ºC. ^1^H-NMR δ (ppm): 0.95 (t, *J =* 7.40 Hz, 6H, **CH_3_**-CH_2_-CH_2_-CH_2_-O), 1.49 (m, 4H,CH_3_-**CH_2_**-CH_2_-CH_2_-O), 1.72 (q, *J =* 6.63 Hz, 4H, CH_3_-CH_2_-**CH_2_**-CH_2_-O), 2.93 (s, 4H, NCH_2_CH_2_N), 4.07 (s, 4H, Ar-CH_2_-N), 4.32 (t, *J =* 6.61 Hz, 4H, CH_3_-CH_2_-CH_2_-**CH_2_**-O), 4.95 (s, 4H, O-CH_2_-N), 6.78 (d, 2H, *J* = 8.3 Hz, Ar-H), 7.68 (d, 2H, *J* = 2.0 Hz, Ar-H), 7.80 (dd, 2H, *J* = 8.3 Hz, *J* = 2.0 Hz, Ar-H). ^13^C-NMR δ (ppm): 13.8, 19.3, 30.9, 49.7, 50.3, 51.8, 83.4, 116.4, 119.5, 122.8, 129.6, 129.7, 158.2, 162.2.

### 3.3. General procedure for reduction of BISBOAs

Sodium borohydride (3.0 mmol, 0.11 g) was added to a solution of the appropriate benzoxazine (1 mmol) in ethanol (15 mL), and the mixture was stirred magnetically for 30 min at room temperature. The progress of the reaction was monitored by thin-layer chromatography (TLC). After completion of the reaction, the mixture was poured into ice-cold water, neutralized with ammonium chloride (12 mL), and extracted with CHCl_3 _(3 × 10 cm^3^). The combined extracts were dried over anhydrous Na_2_SO_4 _and evaporated. The solid obtained was purified by recrystallization from ethanol to the desired products **6a-h**.

*N,N´-bis(2-hydroxy-5-fluorobenzyl)-N,N´-dimethylethane-1,2-diamine* (**6a**). White solid, yield 38%, m.p. 110–112 ºC. IR: 3432 cm^-1^ (O-H), 2849 cm^-1^ (N-CH_3 _str.) ^1^H-NMR δ (ppm): 2.28 (s, 6H, *H_3_C-N*), 2.65 (s, 4H, *NCH_2_CH_2_N*), 3.65 (s, 4H, *Ar-CH_2_-N*), 6.65 (dd, 2H, *J* = 2.0 Hz, *J* = 24.4 Hz, Ar-H), 6.76 (dd, 2H, *J* = 8.7 Hz, *J* = 4.7 Hz, Ar-H), 6.86 (dd, 2H, *J* = 8.4 Hz, *J* = 2.4 Hz, *J* = 16.8 Hz). ^13^C-NMR δ (ppm): 41.7, 53.9, 61.4, 114.9, 115.2, 116.9, 122.4, 153.6, 156.0. Elem. anal. calcd. for C_18_H_22_F_2_N_2_O_2_: C 70.58%, H 6.59%, N 8.33%; found C 70.39%, H 6.54%, N 8.39%.

*N,N´-bis(2-hydroxy-5-chlorobenzyl)-N,N´-dimethylethane-1,2-diamine* (**6b**). White solid, yield 61%, m.p. 172–174 ºC. IR: 3434 cm^-1^ (O-H), 2849 cm^-1^ (N-CH_3 _str.). ^1^H-NMR [400 MHz, δ (ppm), CDCl_3_]: 2.21 (s, 6H, H_3_C-N), 2.58 (s, 4H, NCH_2_CH_2_N), 3.59 (s, 4H, Ar-CH_2_-N), 6.70 (d, 2H, *J* = 8.8 Hz, Ar-H), 6.87 (d, 2H, *J* = 2.4 Hz, Ar-H), 7.05 (dd, 2H, *J* = 8.8 Hz, *J* = 2.4 Hz, Ar-H). ^13^C-NMR [100 MHz, δ(ppm), CDCl_3_]: 40.6, 52.8, 60.2, 116.5, 121.9, 122.6, 127.1, 127.7, 155.3. Elem. anal. calcd. for C_18_H_22_Cl_2_N_2_O_2_: C 58.54%, H 6.00%, N 7.59%; found C 58.29%, H 5.84%, N 7.63%.

*N,N´-bis(2-hydroxy-5-bromobenzyl)-N,N´-dimethylethane-1,2-diamine* (**6c**). White solid, yield 40%, m.p. 181–183 ºC. IR: 3432 cm^-1^ (O-H), 2850 cm^-1^ (N-CH_3 _str.). ^1^H-NMR δ (ppm): 2.23 (s, 6H, H_3_C-N), 2.65 (s, 4H, NCH_2_CH_2_N), 3.58 (s, 4H, Ar-CH_2_-N), 6.73 (d, 2H, *J* = 8.8 Hz, Ar-H), 7.08 (d, 2H, *J* = 2.4 Hz, Ar-H), 7.05 (dd, 2H, *J* = 8.8 Hz, *J* = 2,4 Hz, Ar-H). ^13^C-NMR δ (ppm): 41.5, 53.7, 61.1, 110.7, 117.9, 123.3, 130.9, 131.6, 156.8. Elem. anal. calcd. for C_18_H_22_Br_2_N_2_O_2_: C 47.18%, H 4.84%, N 6.11%; found C 47.19%, H 4.54%, N 6.08%.

*N,N´-bis(2-hydroxy-5-iodobenzyl)-N,N´-dimethylethane-1,2-diamine* (**6d**). White solid, yield 59%, m.p. 170–171 ºC. IR: 3431 cm^-1^ (O-H), 2849 cm^-1^ (N-CH_3 _str.). ^1^H-NMR δ (ppm): 2.27 (s, 6H, H_3_C-N), 2.64 (s, 4H, NCH_2_CH_2_N), 3.64 (s, 4H, Ar-CH_2_-N), 6.62 (d, 2H, *J* = 8.4 Hz, Ar-H), 7.12 (d, 2H, *J* = 2.0 Hz, Ar-H), 7.36 (dd, 2H, *J* = 8.4 Hz, *J* = 2,0 Hz, Ar-H). ^13^C-NMR δ (ppm): 41.5, 53.7, 60.9, 80.4, 118.5, 124.0, 136.8, 137.6, 157.6. Elem. anal. calcd. for C_18_H_22_I_2_N_2_O_2_: C 39.15%, H 4.02%, N 5.07%; found C 38.96%, H 3.99%, N 5.03%.

*N,N´-bis(2-hydroxy-5-methoxycarbonylbenzyl)-N,N´-dimethylethane-1,2-diamine* (**6e**). White solid, yield 36%, m.p. 157–159 ºC. IR: 3433 cm^-1^ (O-H), 2848 cm^-1^ (N-CH_3 _str.), 1704 cm^-1^ (C=O). ^1^H-NMR δ (ppm): 2.29 (s, 6H, CH_3_-N), 2.68 (s, 4H, NCH_2_CH_2_N), 3.75 (s, 4H, Ar-CH_2_-N), 3.87 (s, 6H, CH_3_-O), 6.85 (d, *J =* 8.50 Hz, 2H, Ar-H), 7.70 (d, *J =* 1.48 Hz, 2H, Ar-H), 7.88 (dd, *J =* 8.47 Hz, *J* = 1.94 Hz, 2H, Ar-H). ^13^C-NMR δ (ppm): 41.6, 51.8, 53.8, 61.4, 116.2, 121.0, 121.2, 130.5, 131.1, 162.4, 166.9. Elem. anal. calcd. for C_22_H_28_N_2_O_6_: C 63.45%, H 6.78%, N 6.73%; found C 63.37%, H 6.54%, N 6.63%.

*N,N´-bis(2-hydroxy-5-ethoxycarbonylbenzyl)-N,N´-dimethylethane-1,2-diamine* (**6f**). White solid, yield 66%, m.p. 114 ºC. IR: 3449 cm^-1^ (O-H), 2849 cm^-1^ (N-CH_3 _str.), 1703 cm^-1^ (C=O). ^1^H-NMR δ (ppm): 1.39 (t, *J =* 8.00 Hz, 6H, **CH_3_**-CH_2_-O), 2.31 (s, 6H, CH_3_-N), 2.70 (s, 4H, NCH_2_CH_2_N), 3.77 (s, 4H, Ar-CH_2_-N), 4.34 (q, *J =* 8.00 Hz, 4H, CH_3_-**CH_2_**-O), 6.87 (d, *J =* 8.00 Hz, 2H, Ar-H), 7.72 (d, *J =* 2.00 Hz, 2H, Ar-H), 7.91 (dd, *J =* 8.00 Hz, *J* = 2.00 Hz, 2H, Ar-H). ^13^C-NMR δ (ppm): 13.6, 41.6, 53.8, 60.6, 61.5, 116.1, 121.1, 121.4, 130.4, 131.0, 162.2, 166.4. Elem. anal. calcd. for C_24_H_32_N_2_O_6_: C 64.85%, H 7.26%, N 6.30%; found C 64.72%, H 7.15%, N 6.08%.

*N,N´-bis(2-hydroxy-5-propoxycarbonylbenzyl)-N,N´-dimethylethane-1,2-diamine* (**6g**). White solid, yield 70%, m.p. 123–125 ºC. IR: 3450 cm^-1^ (O-H), 2844 cm^-1^ (N-CH_3 _str.), 1700 cm^-1^ (C=O). ^1^H-NMR δ (ppm): 1.02 (t, *J =* 7.42 Hz, 6H, **CH_3_**-CH_2_-CH_2_-O), 1.77 (m, *J =* 7.5 Hz, 4H, CH_3_-**CH_2_**-CH_2_-O), 2.29 (s, 6H, CH_3_-N), 2.69 (s, 4H, NCH_2_CH_2_N), 3.76 (s, 4H, Ar-CH_2_-N), 4.23 (t, *J =* 6.66 Hz, 4H, CH_3_-CH_2_-**CH_2_**-O), 6.85 (d, *J =* 8.50 Hz, 2H, Ar-H), 7.71 (d, *J =* 1.48 Hz, 2H, Ar-H), 7.90 (dd, *J =* 8.49 Hz, *J* = 2.02 Hz, 2H, Ar-H). ^13^C-NMR δ (ppm): 10.5, 22.2, 41.6, 53.8, 61.5, 66.2, 116.1, 121.0, 121.5, 130.4, 131.1, 162.3, 166.5. Elem. anal. calcd. for C_26_H_36_N_2_O_6_: C 66.08%, H 7.68%, N 5.93%; found C 65.89%, H 7.54%, N 5.78%.

*N,N´-bis(2-hydroxy-5-butoxycarbonylbenzyl)-N,N´-dimethylethane-1,2-diamine* (**6h**). White solid, yield 68%, m.p. 117–119 ºC. IR: 3449 cm^-1^ (O-H), 2850 cm^-1^ (N-CH_3 _str.), 1704 cm^-1^ (C=O). ^1^H-NMR δ (ppm): 0.97 (t, *J =* 7.38 Hz, 6H, **CH_3_**-CH_2_-CH_2_-CH_2_-O), 1.47 (m, 4H, CH_3_-**CH_2_**-CH_2_-CH_2_-O), 1.73 (q, *J =* 6.73 Hz, 4H, CH_3_-CH_2_-**CH_2_**-CH_2_-O), 2.30 (s, 6H, CH_3_-N), 2.68 (s, 4H, NCH_2_CH_2_N), 3.76 (s, 4H, Ar-CH_2_-N), 4.28 (t, *J =* 6.61 Hz, 4H, CH_3_-CH_2_-CH_2_-**CH_2_**-O), 6.85 (d, *J =* 8.50 Hz, 2H, Ar-H), 7.70 (d, *J =* 1.48 Hz, 2H, Ar-H), 7.89 (dd, *J =* 8.49, *J* = 2.02 Hz, 2H, Ar-H). ^13^C-NMR δ (ppm): 13.8, 19.3, 30.9, 41.6, 53.8, 61.5, 64.5, 116.1, 121.1, 121.5, 130.4, 131.1, 162.3, 166.5. Elem. anal. calcd. for C_28_H_40_N_2_O_6_: C 67.18%, H 8.05%, N 5.60%; found C 67.02%, H 7.94%, N 5.43%.

## 4. Conclusions

In summary, we have found a novel synthetic approach for the synthesis of tetrahydrosalens. The features of the present method include the ready availability of the starting materials, the mild reaction conditions, and the simplicity of the workup. Because the substitution pattern on the phenols may be varied, this simple methodology should be useful for the preparation of a variety of *N,N´*-dimethylated-tetrahydrosalens. Furthermore, the simplicity of the operations involved represents a good prerequisite for large scale applications.
